# Curcumin Successfully Inhibited the Computationally Identified CYP2A6 Enzyme-Mediated Bioactivation of Aflatoxin B1 in Arbor Acres broiler

**DOI:** 10.3389/fphar.2017.00143

**Published:** 2017-03-21

**Authors:** Ishfaq Muhammad, Xiaoqi Sun, He Wang, Wei Li, Xinghe Wang, Ping Cheng, Sihong Li, Xiuying Zhang, Sattar Hamid

**Affiliations:** Department of Basic Veterinary Science, College of Veterinary Medicine, Northeast Agricultural UniversityHarbin, China

**Keywords:** aflatoxin-B1, AA broilers, CYP2A6, liver, curcumin

## Abstract

Cytochrome P450 enzymes are often responsible for the toxic and carcinogenic effects of toxicants, such as aflatoxin B1 (AFB1). The human hepatic CYP2A6 enzyme mediates the oxidative metabolism of several procarcinogens. In this study, we characterized a partial sequence of CYP2A6 gene from Arbor Acres (AA) broiler and studied its role in AFB1 bioactivation. Moreover, the effect of curcumin on CYP2A6 is illustrated. Six groups of AA broiler were treated for 28 days including the control group (fed only basal diet), curcumin alone-treated group (450 mg/kg feed), the group fed AFB1-contaminated feed (5 mg/kg feed) plus the low (150 mg), medium (300 mg) or high (450 mg) of curcumin, and the group fed AFB1-contaminated diet alone (5 mg/kg feed). After the end of treatment period, liver samples were collected for different analyses. The results revealed that the histopathological examination showed clear signs of liver toxicity in AA broliers in AFB1-fed group, but curcumin-supplementation in feed prevented partially AFB1-induced liver toxicity. Liver and body weights were recorded to study the AFB1 harmful effects. We noted an obvious increase in liver weight and decrease in body weight in AFB1-fed group. But, the administration of curcumin partially ameliorated the increase in liver weight and decrease in body weight in a dose-dependent manner. The results (RT-PCR and Elisa) revealed that mRNA and protein expression level enhanced in AFB1-fed group. Consistently, CYP2A6 enzyme activity also increased in AFB1-fed group, suggesting that AA broiler CYP2A6 actively involved in bioactivation of AFB1. However, curcumin treatment inhibited CYP2A6 at mRNA and protein levels in AFB1 treated AA broiler in a dose-dependent manner. Maximum inhibition of liver CYP2A6 enzyme activity in AA broiler has been achieved at a dose of 450 mg/kg curcumin. This is the first study identifying and confirming the role of CYP2A6 enzyme in AFB1 bioactivation in AA broiler liver (*in vivo*), and the hepatoprotective role of curcumin via inhibiting CYP2A6 expression and enzyme activity. The study contributed to identify an important CYP enzyme involved in AFB1 bioactivation in broilers and thus could pave the way for the prevention of the harmful effects of AFB1 in broilers.

## Introduction

Cytochrome P450 (CYP) enzymes metabolize various exogenous and endogenous compounds (Nelson et al., 1996). It is well understood that several CYP isoforms involved in carcinogenesis process by metabolically activating procarcinogens to its highly active, DNA-binding metabolites (Gonzalez and Gelboin, 1994). Aflatoxins are well known hepatotoxic, hepatocarcinogenic and mutagenic agents for both animals and humans. These are the food-borne secondary toxic fungal metabolites produced during the growth of *Aspergillus flavus* and *A. parasiticus* (Verma, 2004). Aflatoxin B1 (AFB1) is considered the most biologically active form. It is being increasingly identified that long-term consumption of low levels of this mycotoxin can be harmful to human and animal health. AFB1 is concerned with hepatotoxicity, genotoxicity, carcinogenicity, immunosuppression and other undesirable effects in many animal species including poultry (Richard, 2007; Rawal et al., 2010a). It could be bioactivated in liver by cytochrome P450 enzymes (CYP450) to a highly reactive electrophilic and unstable metabolite that is able to react with cellular macromolecules causing genotoxicity (reacts with DNA) and cytotoxicity (react with proteins) (Doi et al., 2002; Cervino et al., 2007). Some earlier studies reported that the main CYP enzymes responsible for the biotransformation of AFB1 into aflatoxin-8,9-epoxide (AFBO) in mammals were CYP2A6, CYP1A2 and CYP3A4. (Gallagher et al., 1996; Guengerich et al., 1996, 1998). Kamdem et al. (2006) identified in human that CYP3A4 is the main enzyme responsible for AFB1 bioactivation into AFQ1 and aflatoxin-8,9-epoxide, and the main metabolite of AFB1 was AFQ1 (63–95%) (Kamdem et al., 2006). The enzymatic activities of CYP1A1, CYP1A2, CYP2A6 and CYP3A4 have been detected earlier in chickens and quails liver microsomes. A strong correlation has been demonstrated between the activities of CYP2A6 and CYP1A1 (to a lesser extent) with the biotransformation of AFB1 into AFBO. The AFBO production rate is higher in liver microsomes of quail compared with chickens which could partially explain the higher sensitivity of quail to aflatoxins (Diaz et al., 2010). Rawal et al. (2010b) reported that chicken CYP3A37 bioactivate AFB1 to aflatoxin-8,9-epoxide and AFQ1 and was suggested to play a crucial role in avian xenobiotic metabolism relative to other CYP3A, and chicken CYP3A37 isoform shows 60% similarity with human CYP1A4 (Ourlin et al., 2000). Sensitivity to AFB1 by poultry species not only related to biotransformation of AFB1 by Phase I enzymes but also to conjugation of AFBO with glutathione (Phase II enzymes) (Diaz et al., 2010). The highly electrophilic metabolite (aflatoxin-8,9-exo-epoxide) of AFB1 binds to guanine residues in nucleic acids causing mutations in DNA and leading to liver cancer in ducks, humans, and primates (Eaton and Gallagher, 1994; Verma, 2004; Do and Choi, 2007). CYP2A6 is the primary P450 involved in catalyzing nicotine to cotinine in human (Messina et al., 1997). The increased expression of CYP2A6 enzyme has been involved in greater risk of liver cancer in human populations where aflatoxin exposure is common and over expression of CYP2A6 protein was associated with cirrhosis and chronic inflammation (Kirby et al., 1993). CYP2A6 enzyme also catalyzes several human procarcinogens and promutagens, including the carcinogen AFB1 (Yun et al., 1991; Salonpaa et al., 1993). Thus, it is suggested that CYP2A6 may also serve as indicator for hepatic toxicity. Moreover, it has been previously reported that CYP2A6 was responsible for the bioactivation of AFB1 into AFBO in both quail and chicken hepatic microsomes (*in vitro* study) (Diaz et al., 2010). However, only limited information is available regarding chicken CYP2A6 and whether broiler has CYP2A6 and its role in bioactivation of AFB1 has been seldom reported.

Only very few medicinal plants bewitch the interest of scientists, and one such plant is *Curcuma longa Linn.* Turmeric, a yellow product in the rhizomes of *Curcuma longa Linn* possess anti-inflammatory activity (Srimal and Dhawan, 1973; Mukhopadhyay et al., 1982), and antioxidant properties (Sharma, 1976; Araujo and Leon, 2001). It has been considered as a potential third generation cancer chemopreventive agent by oncologists (Lin and Lin-Shia, 2001) and showed highly protective effects against AFB1 (Soni et al., 1997). It is reported earlier that curcumin modulates the activity of microsomal enzymes (phase-I enzymes) and has the potential to control chemical carcinogenesis (Firozi et al., 1996). It has been shown previously that turmeric pretreatment decreased the Benzo[a]Pyrene-induce phase-I enzymes such as CYP1A1/1A2 activity in tissues of rat (Thapliyal and Maru, 2001) and down-regulation of CYP1A1 and CYP2H1 gene in chickens (Yarru et al., 2009a). So far, whether could curcumin, an antioxidant found in turmeric (*Curcuma longa*) powder, modulate the activity of CYP2A6 of broiler fed AFB1 is unknown.

Based on the postulated role of the chicken CYP2A homolog in the sensitivity toward AFB1, the importance of CYP2A6 in the toxicology of AFB1 in humans and animals, and its genetic implication in hepatic cellular cancer (HCC), lung cancer, colorectal cancer etc., we hypothesize that the broilers has the homologue of human CYP2A6 that may bioactivate AFB1. The objectives of our study are: (1) to identify liver CYP2A6 ortholog in chicken liver that has AFB1 oxidizing activity and partially characterize this gene from AA broiler liver; (2) to investigate whether curcumin supplementation of AA broiler diets could affect the transactivation of CYP2A6 enzyme.

## Materials and Methods

### Chemicals and Reagents

Pure AFB1 (purity ≥ 99.0%) was purchased from Sigma–Aldrich (St. Louis, MO, USA). Absolute ethanol (99.0%), methanol, isopropanol, chloroform, agarose G-10, sodium chloride (NaCl) and glycine were bought from Tian Li Company Ltd. (Tianjin, China). Curcumin (2.5%) was bought from Shengxing chemical product company Ltd. (Henan, China). Hematoxylin was purchased from Guangzhou Chemical Reagent Company (China) and eosin was purchased from Nanjing Chemical Reagent Factory (China).

### Arbor Acres (AA) Broiler and Experimental Protocol

Healthy AA broiler chickens were purchased from an authorized (Registration number; 230108799294096) commercial hatchery (Yi Nong, Harbin, Heilongjiang, China). The broilers were weighed and assigned to different cages. Proper bedding material provided and divided into six groups. Each group provided randomly with 20 chicks. The AA broilers were kept on 12 h light and 12 h dark, fed under standard managemental conditions and AA broiler mortality was recorded during the trial period. All of the experiments were conducted under the supervision of the Harbin Veterinary Research Institute of the Chinese Academy of Agricultural Sciences in accordance with animal ethics guidelines and approved protocols. The Harbin Veterinary Research Institute Animal Ethics Committee approval number was SYXK (Hei) 2012-2067. Aflatoxin binder free diet was purchased from Shen Nong Feed Company (Harbin, PR China). Six dietary treatments included (1) CG; basal diet supplemented with 0 mg AFB1/kg feed and 0 mg curcumin/kg feed (control group, CG) (2) CCG; basal diet supplemented with 450 mg curcumin/kg feed (curcumin control group, CCG) (3) R1G; basal diet supplemented with 5.0 mg AFB1/kg feed and 150 mg curcumin/kg feed (4) R2G; basal diet supplemented with 5.0 mg AFB1/kg feed and 300 mg curcumin/kg feed (5) R3G; basal diet supplemented with 5.0 mg AFB1/kg feed and 450 mg curcumin /kg feed (6) AFB1 G; basal diet contaminated with 5.0 mg AFB1/kg feed (AFB1 group). 10 mg of AFB1 was first dissolved in a known volume of methanol (30 ml). Then, the AFB1-methanol solution was evenly sprayed over the feed and thoroughly mixed to obtain the required concentration of AFB1 (5 mg AFB1/kg feed), and finally the feed air-dried at 37°C. AFB1 is toxic and should be handled with great care to avoid contact/inhalation. It should only be handled in a fume hood while wearing gloves, lab coat, and goggles (to protect eyes) and all contacted materials should be properly disposed of in an environmentally safe manner.

### Sample Collection, Body Weight, Liver Weight and Liver Index

After receiving the diets for 28 days, AA broiler chickens were sacrificed by cardiac puncture following euthanasia with sodium pentobarbital in an antiseptic environment. Body weights were recorded before euthanasia. The entire liver was collected and weight was recorded. The liver index reflected the liver weight/body weight ratio. A piece of liver tissue (10–14 g) was stored at -80°C for further analysis.

### Histopathological Examination

Liver histopathology was evaluated via hematoxylin and eosin (HE) staining (Wang X.H et al., 2016). In brief, fresh liver tissue pieces (1 cm × 1 cm) were fixed in 10% **formalin** for more than 24 h, after processed in a series of graded ethanol and dimethyl benzene, then embedded in paraffin. Tissue sections (4 μm) were sliced and mounted on glass slides. The slides were then stained with HE for histopathological examination and were examined under a light microscope (Nikon E100, 40X magnification).

### Primer Designing for CYP2A6 and Sequencing of the PCR Product

As CYP2A6 gene sequence is unknown in AA broiler but it was predicted previously (Gonzalo and Hansen, 2011). Degenerate primers were designed for CYP2A6 gene on the bases of the sequence alignments (ClustalW) of homologous genes of Human (CYP2A6; NP_000753.3, CYP2A7; NP_000755.2), Rhesus monkey (CYP2A24; NP_001035305.1), Mouse (CYP2A5; NP_031838.2), Rat (CYP2A3; NP_036674.1), Frog (CYP2A6; NP_001010998.1) and *Caenorhabditis elegans* (CYP36A1; NP_492267.1) by Codehop program^1^. The best score primers were selected for PCR. The forward and reverse sequence of the primer are TGTGCGCCGTCATC cangarrtnya-3′ and acrranccnct CCCGGACCGGGC-5′ respectively. The optimum PCR conditions were as follows: initial denaturation step at 95°C for 4 min; 95°C for 10 s (denaturation); 58°C for 10 s (annealing); 35 cycles at 72°C for 25 s (elongation); and 72°C for 5 min (final extension step)^2^. The PCR product was then cloned under UV light and sent for sequencing to the Sangon Biotech (Shanghai, China). The sequencing result was blast in National Centre for Biotechnology Information (NCBI) and a phylogenetic tree was constructed.

### Extraction of RNA and Real-Time PCR Examination

Hepatic total RNA was isolated using the TRIzol extraction method according to the manufacturer’s instructions (Invitrogen Inc., Carlsbad, CA, USA). The RNA quality was verified by evaluating the optical density at 260 and 280 nm (Wang J et al., 2016). A high quality RNA was prepared, reverse transcribed to cDNA using a first strand cDNA synthesis kit (Toyobo, Co., Ltd. Osaka, Japan) and was subjected to RT-PCR containing specific primers for the gene CYP2A6 and glyceraldehyde-3-phosphate dehydrogenase (GAPDH) as the loading control gene in the LightCycle instrument (Roche). The CYP2A6 forward and reverse primer used for RT-PCR are CTGCAGAGAATGGCATGAAG and CCTGCAAGACTGCAAGGAA respectively. The kit used for RT-PCR were purchased from Toyobo Company Ltd. (Japan). Data were analyzed and quantified using the 2^-ΔΔ^Ct method (Livak and Schmittgen, 2001).

### Measurement of CYP2A6 Enzyme Activity

Microsomal CYP2A6 activity was determined by measuring the coumarin 7-hydroxylase (COH) level using the method as described by Li et al. (2013). Briefly, a piece of liver tissue was weighed (150–200 mg), cut with scissor and lysed in homogenization buffer (pH 7.4), and microsomal extracts were extracted by a series of differential centrifugations at 4°C using Beckman ultra-high speed centrifuge. Then 0.1 mg of microsomal protein was incubated with 100 mM coumarin and NADPH for 15 min at 37°C in a shaking incubator. The excitation and emission wavelength for Fluorescence was set at 355 and 460 nm respectively using a Fluoroskan Ascent (Thermo Fisher Scientific, Shanghai, China).

### CYP2A6 Specific Protein Linked Immunosorbant Assay (ELISA)

The monoclonal CYP2A6 ELISA kit (competitive enzyme immunoassay) bought from Bio-Source Company Ltd (Southern California, San Diego, CA, USA). The assay was done according to the manufacturer instructions. Liver tissue samples were weighed (300–350 mg), rinsed in ice-cold PBS (pH 7.0 – 7.2) to remove excess blood before homogenization. The tissue were minced and then homogenized in a tissue homogenizer (Shanghai, China) in 500 μl of PBS. The resulting suspension was subjected to ultrasonication and centrifuged for 15 min at 5000 rpm. The supernatant was taken, diluted and assayed immediately. All the samples and standards along with a blank control were run in duplicate and the readings were taken at 450 nm using an iMARK^TM^ microplate reader (Bio-Rad Co., Ltd. Shanghai, China) and the protein concentration was quantified.

### Statistical Analysis

All the assays were performed in triplicates unless otherwise mentioned. Significance of the data were measured by statistical package for social science (SPSS, version 21.0). Differences between groups were analyzed by one way ANOVA followed by Tukey’s test and *p* < 0.05 were considered statistically significant. The results expressed as mean ± standard error. All the graphs with standard error bar were made on SigmaPlot (Version 10.0).

## Results

### AA Broiler Mortality, Health and Phenotypic Effects of AFB1

We have observed no (zero) mortality in all experimental groups during the 28 days trial period. The broiler fed curcumin alone (CCG) showed no significant abnormal signs. Compared to CG and CCG, impaired feathering, lethargy and yellow skin were observed in broiler chickens fed AFB1. These signs disappear with curcumin treatment in all the three groups (R1G, R2G, and R3G). Ruffled feathers were noted in AA broiler chickens in low dose curcumin group (R1G). In order to study the phenotypic effects of AFB1, liver weight and body weight of AA broiler chickens were recorded. The increase in liver weight was statistically significant (*p* < 0.05) in AFB1-fed group compared to CCG, but was not statistically significant relative to CG as shown in **Table 1**. Body weights were also decreased significantly (*p* < 0.05) in AFB1 fed group as compared to control group and curcumin control group, which provide an insight into the mechanism of toxicity. However, the addition of curcumin partially ameliorated the increase in liver weight and decrease in body weight in a dose dependent manner (R1G, R2G, and R3G). It is noted that 450 mg/kg curcumin (R3G) treatment achieved maximum amelioration against AFB1-induced increase in liver weight and decrease in body weight (**Table 1**).

**Table 1:** Liver weight, body weight and liver index.

Treatments	Liver weight (g/bird)	Body weight (g/bird)	Liver index (liver weight/body weight)
CG	22.673 @ 1.543	996.667 @ 20.276ˆa,b,c,d,e,f	0.022803 @ 0.002ˆa,c,f
CCG	18.144 @ 0.859ˆa	876.4 @ 13.71ˆa,b,c,d,f	0.0207 @ 0.001ˆb,c,d,f
R1G	26.238 @ 1.523ˆa	677.6 @ 16.467ˆa,b,c,e	0.0389 @ 0.003ˆa,b,c,e
R2G	23.93 @ 2.65	754.8 @ 26.442ˆa,b,d,f	0.0322 @ 0.004ˆb,d,f
R3G	22.028 @ 0.597ˆb	830 @ 15.093ˆa,c,e,f	0.0266 @ 0.0007ˆc,e,f
AFB1G	29.538 @ 1.914ˆa,b	612 @ 20.271ˆa,b,d,f	0.048 @ 0.0025ˆa, b,d,e,f

*Treatment indicated as CG = Control group (basal diet), CCG = Curcumin control group (450 mg curcumin /kg feed), R1G = 150 mg curcumin + 5.0 mg AFB1/kg feed, R2G = 300 mg curcumin + 5.0 mg AFB1/kg feed, R3G = 450 mg curcumin + 5.0 mg AFB1/kg feed, AFB1 G = 5.0 mg AFB1/kg feed. Values represented as mean ± standard error (SE). The value (mean ± SE) of control group shows three observations (*n* = 3), while the remaining values shows five observations per group (*n* = 5). Means with different letters statistically significant according to Tukey’s HSD test (*p* ≤ 0.05).*

### Histopathological Examination

**FIGURE 1:**
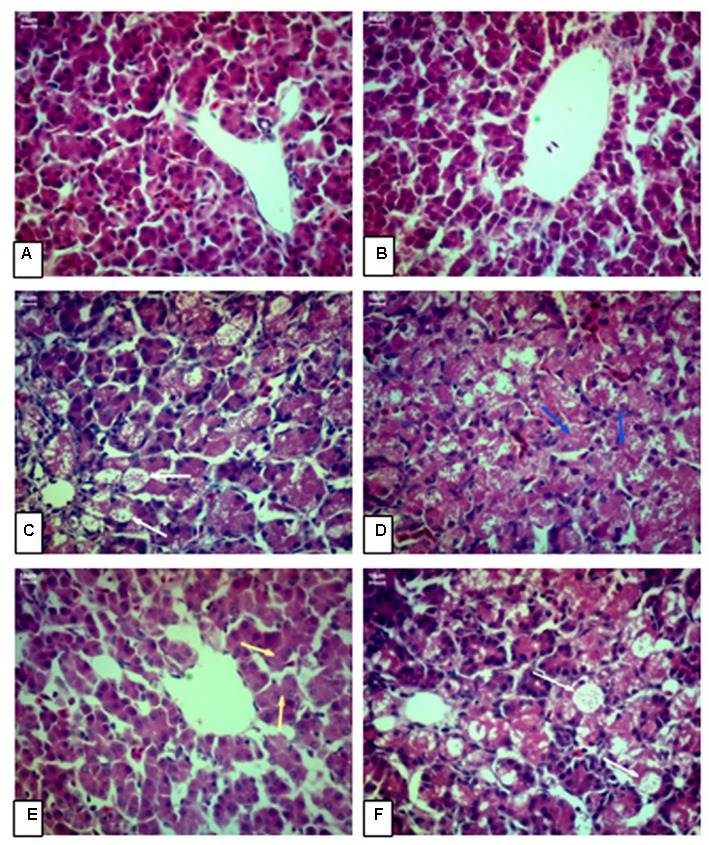
**Chicken hepatic pathology.** Liver sections were stained by hematoxylin and eosin and examined by light microscopy (40X magnification). **(A)** Control group (CG; fed only basal diet), **(B)** Curcumin control group (CCG; 450 mg curcumin/kg feed), **(C)** Low dose curcumin group (R1G; 150 mg curcumin + 5.0 mg AFB1/kg feed), **(D)** Medium dose curcumin group (R2G; 300 mg curcumin + 5.0 mg AFB1/kg feed), **(E)** High dose curcumin group (R3G; 450 mg curcumin + 5.0 mg AFB1/kg feed), **(F)** AFB1 group (AFB1 G; 5.0 mg AFB1/kg feed). (H&E, scale bar = 10 μm).

**Figure 1** shows the photomicrographs of HE-stained liver sections of AA broilers. There were no microscopic lesions in the livers of broilers received control diet (**Figure 1A**) and broilers fed control diet plus curcumin (**Figure 1B**). AFB1 group (**Figure 1F**), and the AFB1 plus low dose of curcumin group (**Figure 1C**) showed a severe liver damage, vacuolar degeneration, fatty degeneration and ballooning of the hepatocytes relative to control group (**Figure 1A**). As compared to control group (**Figure 1A**), medium dose group (**Figure 1D**) showed moderate congestion and vacuolar degeneration. While the high dose curcumin group (**Figure 1E**) showed granular degeneration in hepatocytes relative to control group (**Figure 1A**). Thus, these results showed that low dose curcumin had no effect in the prevention of liver damage induced by AFB1, while the medium and high dose curcumin can prevent liver injury partially. As the amount of curcumin increased in the diet, the amelioration effect of curcumin becomes more apparent, especially with the high dose (450 mg/kg) curcumin-treated group (**Figure 1E**).

### Sequencing of PCR Product and Construction of Phylogenetic Tree

**FIGURE 2:**
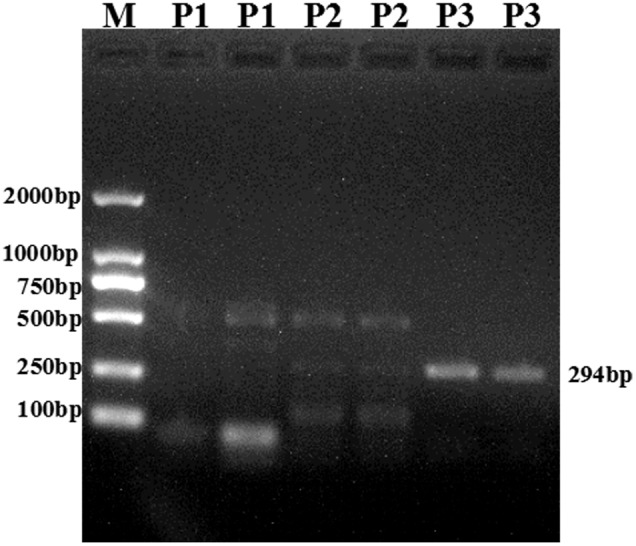
**The PCR product was run and visualized on 1.0% agarose gel and a clear band (294 bp) was obtained for primer 3 (P3).** The Marker (M) 2 Kbp was loaded in Lane1, while the three different PCR products were loaded in the consecutive lanes (P1; Lane 2, 3 P2; Lane 4, 5 and P3; Lane 6, 7). The molecular weight (bp) corresponding to the DNA ladder is indicated on the left side.

**FIGURE 3:**
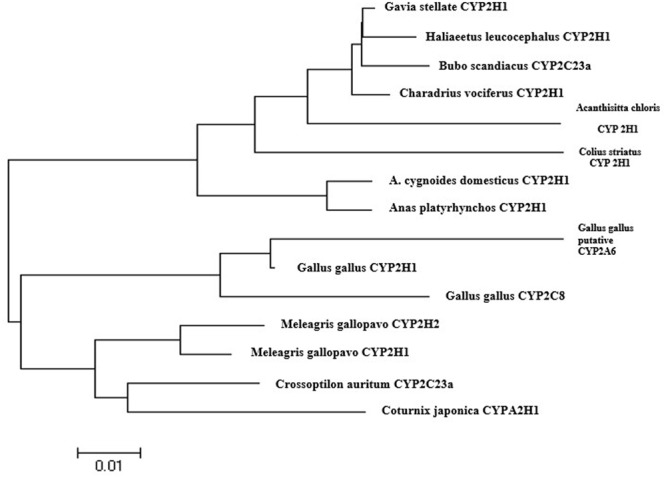
**The nucleotide sequence for the chicken CYP2A6 gene has been deposited in the GenBank database under GenBank accession number *KX687985*.** A phylogenetic tree was generated by the neighbor-joining method using distance relationships by using MEGA4 software. The genes accession numbers selected for the phylogenetic tree are as follows: *Gallus gallus* CYP2H1 like (NM_001001616.1), *Gallus gallus* CYP2C8 (NM_001001757.1), *Coturnix japonica* cytochrome P450 2H1-like (XR_001556094.1), *Meleagris gallopavo* cytochrome P450 2H1-like (XM_010714529.1), *Crossoptilon auritum* CYP2C23a (dbj| AB795991.1), *Anser cygnoides* domesticus CYP2H1-like (XM_013178097.1), *Meleagris gallopavo* CYP2H2 (XM_010714446.1), *Anas platyrhynchos* CYP2H1 (XM_013104761.1), *Charadrius vociferus* CYP2H1-like (XM_009888733.1), *Gavia stellata* CYP2H1-like (XM_009819531.1), *Bubo scandiacus* CYP2C23a (dbj| AB795985.1), *Colius striatus* cytochrome P450 2H1-like (XM_010203599.1), *Haliaeetus leucocephalus* CYP2H1-like (XM_010563515.1), *Acanthisitta chloris* CYP450 2H1-like (XM_009081538.1), *Gallus gallus* putative CYP2A6 (KX687985).

As chicken CYP2A6 gene nucleotide sequence was unknown but this gene was predicted in earlier studies that the putative avian CYP2A6 ortholog may be the main CYP450 enzyme responsible for the bioactivation of AFB1 into its epoxide form in poultry species (Gonzalo and Hansen, 2011). In this study, we identified and characterized a partial sequence of AA broiler CYP2A6 gene by designing degenerate primers as stated in materials and methods. The PCR product (**Figure 2**) was sequenced and blast in NCBI. The nucleotide sequence for the putative broiler CYP2A6 gene has been deposited in the GenBank database under GenBank accession number *KX687985*. Sequence alignment of this sequence shows similarity to mouse CYP2A5 isoform (50%) and human CYP2A6 (49%). The phylogenetic tree was made based on the nucleotide sequence of putative broiler CYP2A6 and the CYP2 family enzymes, selected for the phylogenetic tree which shows a similarity more than 85% according to blast results. The nucleotide sequences were aligned using ClustalW and employed for model selection and construction of maximum likelihood tree using MEGA4 (**Figure 3**). The tree indicates that the characterized sequence is a gene of CYP2 family.

### Effect of AFB1, Curcumin and AFB1-Curcumin Diets on CYP2A6 mRNA Expression

**FIGURE 4:**
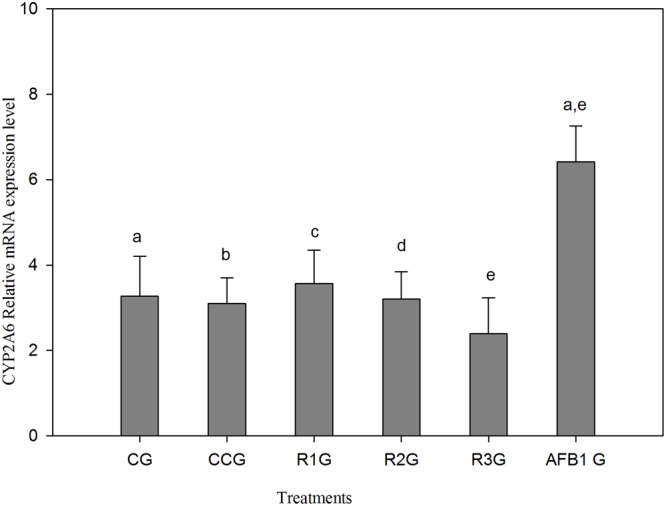
**The values represented as mean ± SE (*n* = 3).** CG, control group (fed only basal diet); CCG, curcumin control group (450 mg curcumin/kg feed); R1G = 150 mg curcumin + 5.0 mg AFB1/kg feed, R2G = 300 mg curcumin + 5.0 mg AFB1/kg feed, R3G = 450 mg curcumin + 5.0 mg AFB1/kg feed, AFB1 G = 5.0 mg AFB1/kg feed. The value of *P* < 0.05 was considered statistically significant. Means with different letters significantly different according to Tukey’s HSD test (*p* ≤ 0.05).

A slight reduction of mRNA expression level has been noted in curcumin alone group (CCG) as compared to CG (**Figure 4**). The group fed AFB1 (AFB1 G) showed significant (*p* < 0.05) up-regulation of CYP2A6 mRNA expression level relative to CG. On the other hand, a dose-dependent reduction of CYP2A6 mRNA expression level has been noted in AFB1-curcumin treated groups (R1G, R2G, and R3G). Thus, the increased expression of CYP2A6 mRNA expression due to AFB1 has been reduced by curcumin and maximum reduction observed at a dose of 450 mg/kg curcumin treatment (R3G).

### Inhibition of CYP2A6 Enzyme Activity by Curcumin

**FIGURE 5:**
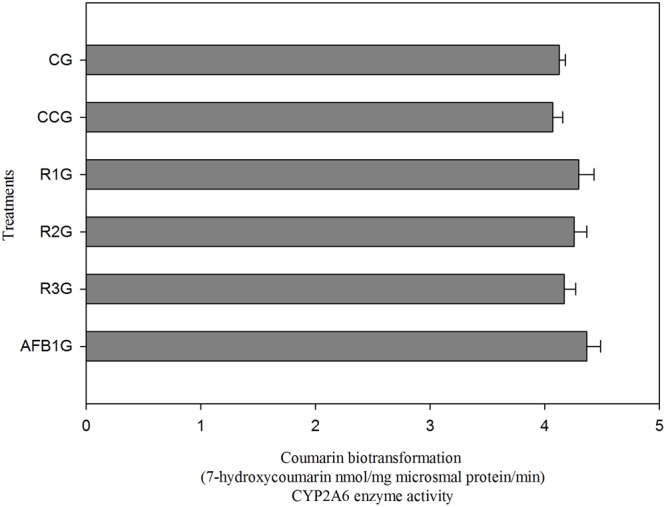
**Enzyme activity of CYP2A6 enzyme (coumarin biotransformation).** The data represented as mean ± SE (*n* = 3). CG, Control group (fed only basal diet); CCG, Curcumin control group (450 mg curcumin/kg feed); R1G = 150 mg curcumin + 5.0 mg AFB1/kg feed, R2G = 300 mg curcumin + 5.0 mg AFB1/kg feed, R3G = 450 mg curcumin + 5.0 mg AFB1/kg feed, AFB1 G = 5.0 mg AFB1/kg feed. The value of *P* < 0.05 was considered statistically significant. Means with different letters significantly different according to Tukey’s HSD test (*p* ≤ 0.05).

**Figure 5** shows the effect of different diets on CYP2A6 enzyme activity. It is observed that CYP2A6 activity is slightly reduced in curcumin alone group (CCG) relative to CG. Compared to CG and CCG, CYP2A6 activity has been significantly (*p* < 0.05) increased in AFB1 fed group (AFB1G), shows its involvement in AFB1 bioactivation. However, the three different doses of curcumin reduced the enzyme activity in a dose-dependent manner (R1G, R2G, and R3G). It is clear that 450 mg/kg curcumin treatment (R3G) effectively (*p* < 0.05) inhibited the enzyme activity of CYP2A6 relative to AFB1 group (AFB1G). Hence, we explored that curcumin has an inhibitory effect on CYP2A6 enzyme activity.

### Effect of AFB1, Curcumin and AFB1-Curcumin Diets on CYP2A6 Protein Expression

**FIGURE 6:**
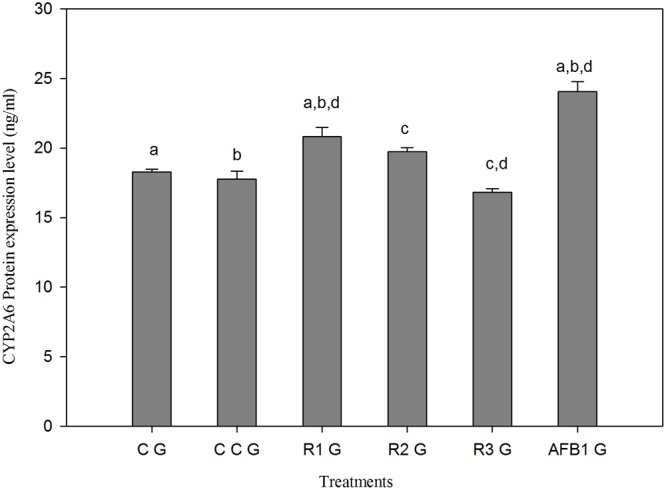
**The effect of AFB1, curcumin and AFB1-curcumin treatment on protein expression of CYP2A6 relative to control group.** The value of *P* < 0.05 was considered statistically significant. The data represented as mean ± SE (*n* = 3). The groups represented as CG = Control group (fed only basal diet), CCG = Curcumin control group (450 mg curcumin/kg feed), R1G = 150 mg curcumin + 5.0 mg AFB1/kg feed, R2G = 300 mg curcumin + 5.0 mg AFB1/kg feed, R3G = 450 mg curcumin + 5.0 mg AFB1/kg feed, AFB1 G = 5.0 mg AFB1/kg feed. Different superscript letters indicate that means significantly different according to Tukey’s HSD test (*p* ≤ 0.05).

Compared to CG, CYP2A6 protein expression reduced in curcumin alone group (CCG), but significantly (*p* < 0.05) increased in AFB1 fed group (AFB1 G) (**Figure 6**). **Figure 6** also shows that the protein expression reduced in a dose-dependent manner with different treatments of curcumin along with AFB1 exposure (R1G, R2G, and R3G). Thus, curcumin effectively decreased the enhanced protein expression of CYP2A6 due to AFB1, and significant reduction (*p* < 0.05) in protein expression is achieved with 450 mg/kg curcumin treatment (R3G) compared with AFB1 fed group (AFB1 G).

## Discussion

This study utilized *in vivo* exposure model to study the harmful effects of AFB1 in AA broiler chickens. The supplementation of curcumin alone in the diet did not have a significant effect on liver weight but significantly (*p* < 0.05) effect the body weight of AA broiler chickens compared to control group. However, as stated in results section, curcumin partially ameliorated the AFB1-induced increase in liver weight and decrease in body weight with the increasing doses of curcumin. The failure of curcumin to further ameliorate the AFB1-induced increase in liver weight and decrease in body weight, it is still unknown. Since, the abnormal changes in liver weight and body weight (compared to control group) is not the only mode of action of AFB1, but there are also some other toxic effects of AFB1 and it has been shown earlier that AFB1 decreased the expression of hepatic genes involved in fatty acid metabolism, energy production, coagulation, detoxification and immunity of male broiler chicks (Yarru et al., 2009b). Our findings of AFB1 harmful phenotypic effects (**Table 1**), are in consistent with earlier studies, that dietary AFB1 in poultry causes increase in liver weight due to lipid accumulation in liver, which results in hepatomegaly and increased liver weight in both chickens and domesticated turkeys (Giambrone et al., 1985; Yarru et al., 2009a; Chen et al., 2014). In addition to these phenotypic changes, hepatic transcriptome analysis showed significant effects on genes involved in cell cycle regulation and NRF2-pathway (Monson et al., 2016). Our histological findings showed severe liver injury in AFB1 fed group. However, the supplementation of curcumin in increasing doses did not completely ameliorate liver injury in this study. These findings are in line with some earlier studies reported about liver injury, toxicity of aflatoxin and the chemopreventive effects of curcumin. (Gowda et al., 2009; Yildirim et al., 2011; Colakoglu and Donmez, 2012).

Diaz et al. (2010) speculated earlier that the putative avian CYP2A6 ortholog may be the main CYP450 enzyme responsible for the bioactivation of AFB1. They found the inhibition of aflatoxin-8,9-epoxide (AFBO) by specific CYP2A6 inhibitor and determined the relationship between CYP2A6 enzyme activity and AFBO formation. In this study, we identified AA broiler CYP2A6 gene and characterized its partial sequence. The phylogenetic tree (**Figure 3**), indicates that the characterized sequence belongs to CYP2 family. It is clear from our results that CYP2A6 is actively involved in AFB1 bioactivation in broiler. Thus it is concluded that CYP2A6 is one of major CYP enzyme that is responsible for the biotransformation of AFB1 in broiler, and these results are in agreement with the study conducted by Diaz et al. (2010) who reported that over expression of chicken CYP2A6 related to aflatoxin-8,9-epoxide (AFBO) production and cause AFB1-DNA adducts. The author also evaluated that CYP2A6 enzyme involved in AFB1 bioactivation in poultry species (duck, quail, turkey, and chicken), and that AFBO production was inhibited by the CYP2A6 inhibitor 8-methoxypsoralen and a significant correlation between coumarin 7-hydroxylation and AFB1 epoxidation had been demonstrated. In addition, CYP1A1, CYP1A2 and CYP3A4 also bio-activate AFB1 into its toxic metabolites in chicken, quail, turkey, and duck liver microsomes (Hansen et al., 2011).

It has been showed previously that curcuminoids yellowish pigments present in rhizomes of turmeric (*Curcuma longa*) alleviate the harmful effects of AFB1 (Lee et al., 2001), inhibit the biotransformation of aflatoxins to their epoxide metabolites (Soni et al., 1997), and reverse the aflatoxin induced liver damage (Soni et al., 1992). Curcumin potently inhibited CYP3A activity in rats (Kim et al., 2015), CYP2D6, CYP2B6, CYP1A2, CYP3A4 and CYP2C9 in human (Appiah-Opong et al., 2007). Our data showed that curcumin has alleviated the enhanced protein and mRNA expression of broiler CYP2A6 enzyme. In addition, we found that the elevated mRNA expression and protein expression of broiler CYP2A6 enzyme due to AFB1, decline with dietary curcumin in a dose-dependent manner. Moreover, curcumin decreased the broiler CYP2A6 enzyme activity in dose-dependent manner, thus reducing the AFB1-DNA adduct formation and inhibited the enzymatic formation of AFB1 metabolites in AA broiler chickens. Hence, we explored that curcumin has an inhibitory effect on the activity of CYP2A6 and thus curcumin would be a valuable drug in preventing the AFB1 toxicity. Maximum inhibition of broiler CYP2A6 mRNA elevated expression had been achieved at high dose (450 mg/kg) curcumin treatment along with AFB1 (5.0 mg/kg) dietary exposure. The protein expression of CYP2A6 showed the same trend as the mRNA expression. This finding is further evaluated by enzyme activity measurement which also showed that high dose curcumin inhibited liver increased CYP2A6 enzyme activity in broiler fed with AFB1 contaminated diet. Similarly, it has been stated earlier that curcumin alleviated the increased expression of CYP2H1 and CYP1A1 in chickens fed AFB1 while increased the expression of certain phase-II enzymes such as GST (Yarru et al., 2009a). Moreover, curcumin further resulted in decreased carcinogen-induced stress and DNA adducts and the inhibitory effects of dietary curcumin on Benzo[a]Pyrene-induced AhR activation and decline in protein expression and enzyme activity of CYP1A1/1A2 (*in vivo*) in mice has also been reported (Garg et al., 2008). In addition, curcumin reduces the levels of superoxide anion formation (Lee et al., 2001), and biotransformation of aflatoxicol in liver (Iqbal et al., 2003). Compared to controls, our data showed that 150 mg/kg and 300 mg/kg curcumin supplementation in diet not fully reduced the increased enzyme activity of broiler liver CYP2A6 along with AFB1 feeding. The mRNA and protein expression results are also in consistent with this finding. Gowda et al. (2009) who demonstrated that dietary supplementation of 74 mg/kg curcumin not completely ameliorated the adverse effects of AFB1 but 222 mg/kg curcumin provided the greatest amelioration in broiler chickens against 1.0 mg/kg dietary AFB1. Our current *in vivo* findings showed that 450 mg/kg dietary curcumin fully alleviated the increased expression of broiler liver CYP2A6 enzyme against 5.0 mg/kg dietary AFB1. Although the molecular mechanism of inhibition is still unknown and needs further studies to be verified. AFB1 is hazardous due to their potential hepatotoxicity and carcinogenicity, and AFB1-epoxides are potent mutagenic agents. Thus, inhibition of phase-I and enhancement of phase-II enzymes in liver appear to play highly significant role in preventing AFB1-induced hepatocellular carcinogenesis and mutagenesis.

## Conclusion

This is the first study to characterize the partial sequence of liver CYP2A6 gene in AA broiler, which may help in cloning and complete molecular characterization of CYP2A6 gene, in future, to further define its role in various drug and toxin induced toxicities. Our findings suggest that curcumin protects liver from AFB1 toxicity, and that higher doses of curcumin negatively modulates the bioactivation of AFB1. Additionally, the effect of curcumin on CYP2A6 enzyme has been demonstrated along with AFB1 exposure. Taken together, our findings may provide an insight into the mechanism of AFB1-induced hepatotoxicity in broiler liver and the preventive effects of curcumin against AFB1.

## Author Contributions

XZ supervised the whole experiments. IM and XS performed the practical work and completed the experiments, HW, WL, XW, PC, and SL provided help during the experiments. SH helped in improving language expression.

## Conflict of Interest Statement

The authors declare that the research was conducted in the absence of any commercial or financial relationships that could be construed as a potential conflict of interest.

## Footnotes

1 http://blocks.fhcrc.org/codehop.html

2 http://www.eeescience.utoledo.edu/Faculty/Sigler/Von_Sigler/LEPR_Protocols_files/PCR.pdf
